# Free Vascularized Fibula Salvage of Failed CPH in Pediatric Sarcoma Patients

**DOI:** 10.1155/2022/6240293

**Published:** 2022-05-09

**Authors:** Giovanna Pires, Whitney D. Moss, Jessica Luo, Ruyan Zhang, Kevin B Jones, Alvin C Kwok, Jayant P Agarwal

**Affiliations:** ^1^Division of Plastic Surgery, Department of Surgery, University of Utah, Salt Lake City, UT, USA; ^2^Department of Orthopedics, University of Utah, Salt Lake City, UT, USA

## Abstract

**Background:**

Due to extended life expectancy and recent improvements in surgical techniques, limb salvage has replaced amputation as the gold standard and is now performed in 90–95% of upper extremity malignancies. However, many of these salvage procedures are associated with significant postsurgical complications. In particular, the clavicula pro humero (CPH) procedure is associated with high rates of nonunion. We present our experience with upper extremity salvage using the free vascularized fibular flap (VFF) after failure or nonunion of the original CPH procedure in the pediatric population.

**Methods:**

Five patients under the age of 18 diagnosed with upper extremity sarcoma who underwent tumor resection with immediate CPH reconstruction complicated with nonunion, and subsequent revision with free VFF were included. Data on patient demographics, oncologic characteristics, surgical procedures, intraoperative details, postoperative complications, and time to graft union were recorded.

**Results:**

Five patients (average age = 8.4 years; range = 5–10 years at surgery date) underwent secondary limb salvage procedure with free VFF reconstruction following failed CPH reconstruction for proximal humeral osteosarcoma (*n* = 4) or Ewing sarcoma (*n* = 1). The mean follow-up was 3.7 years. Complications occurred in five patients (100%), with three patients requiring reoperation (60%). Four patients achieved graft union (average union time = 3.7 months) and successful limb reconstruction. Four patients were alive with no local recurrence of the disease. One patient did not achieve union and was lost to follow-up.

**Conclusion:**

Primary bone tumors in the pediatric population require wide surgical resection, and reconstruction often has high complication rates that can warrant further procedures. A free VFF is a viable option for upper extremity salvage after previously failed reconstructions because it provides vascularized tissue to a scarred tissue bed and allows for the replacement or augmentation of large bony defects.

## 1. Introduction

Primary bone tumors account for 6% of malignancies in the pediatric population younger than 20 years of age, with osteosarcoma (56%) and Ewing sarcoma (34%) being the most predominant [1]. The mainstay of treatment for both malignancies includes multiagent chemotherapy punctuated by wide surgical resection for local control [2]. With the advancement of diagnostic imaging and chemotherapy strategies, the 5-year survival rate for children has increased to 60–79% [3, 4]. Due to the extended life expectancy and improvement in surgical techniques, limb salvage has replaced amputation as the gold standard and is now performed for 90–95% of upper extremity malignancies [2, 5, 6].

The proximal humerus is the fourth most common location for primary bone tumors [2]. In the pediatric population, the tendency of primary malignant tumors to develop near the physeal growth centers of long bones presents unique challenges for reconstruction [2, 5]. Often, the resection involves the humeral head, extensive musculature, rotator cuff tendons, and axillary nerve, which makes the reconstructive goals of a sustainably stable shoulder with maximum mobility difficult to achieve [5]. Due to skeletal immaturity, resection of the physis prevents further growth and can result in the development of limb length discrepancies [5]. Reconstructive options range from metal endoprosthetic implants, cadaveric allografts, and vascularized or nonvascularized autografts [2, 5]. Each option exposes the patient to modest benefits, significant functional challenges, and high rates of complications. This has resulted in a lack of consensus amongst surgeons on the best reconstructive method. Ultimately, the type of reconstruction depends on surgeon preference, experience, patient-specific factors (e.g., age, preference, and functional requirements), and tumor-specific factors (e.g., size, location, and associated soft tissue involvement) [7].

At our institution, clavicula pro humero (CPH) has been offered to young patients with primary bone malignancies in the proximal humerus. CPH uses native anatomy to reconstruct a stable and durable proximal humerus that permits further growth and limits other donor site morbidity [8]. Since the first description of the use of CPH for proximal humerus reconstruction in 1992 by Winkelmann [9], there have been only a handful of case reports and case series assessing surgical outcomes for these patients. These published cases have revealed that CPH can maintain shoulder stability with comparable mobility (average 75° on flexion) and superior Musculoskeletal Tumor Society (MSTS) functionality scores (83%) [10, 11]. However, the published complication rates for this procedure are high, with a substantial number of documented postoperative complications requiring surgical revision (Supplemental Table 1) [8, 12–20]. The largest pediatric case series assessing postoperative outcomes following CPH for proximal humerus reconstruction revealed proximal nonunion requiring revision surgery occurred in five of seven patients (71%) [19]. Due to the expected risks of nonunion and fracture following CPH, it is essential to anticipate possible revision procedures. However, since the CPH procedure itself is not commonly performed given the low frequency of proximal humeral sarcomas, there is a paucity of literature on secondary salvage procedures following CPH failure.

The vascularized fibular flap (VFF) has become the workhorse for segmental bony reconstruction and an option for salvage after failed endoprosthetic, allograft, or other autograft reconstructions. It has the advantages of enhanced rates of biologic incorporation and the ability to thrive in compromised soft tissue environments [7, 21, 22]. While VFF has been described as a primary reconstruction option for long bone sarcomas [23] and a successful salvage procedure for femur reconstruction and allograft failure [24], no reports have been published on the use of VFF as a secondary salvage procedure following failed CPH in pediatric patients. In this study, we retrospectively review all pediatric patients at our institution who underwent secondary salvage using the vascularized fibular flap (VFF) after nonunion of the original proximal humerus reconstruction with the CPH procedure.

## 2. Methods

This retrospective study was exempted by our institution's Institutional Review Board (IRB00118828). Chart review identified patients under the age of 18 diagnosed with upper extremity sarcoma who underwent tumor resection with immediate CPH reconstruction complicated by nonunion, and subsequent revision surgery with free VFF. The following data were recorded: patient demographics, comorbidities, primary diagnosis, malignancy location, presence of metastatic disease, adjuvant therapy, procedure performed, vessel anastomosis, imaging, mortality, limb survival, local recurrence, complications, reoperations, and time to graft union. Graft union time was determined by the visualization of bridging callus or the obscuring of the osteotomy site at the proximal and distal ends of the fibular graft on radiograph interpretation by an orthopedic surgeon on follow-up imaging.

### 2.1. Surgical Technique

All five patients originally underwent an immediate tumor resection and CPH reconstruction by a senior orthopedic surgeon at our institution (Supplemental Table 2). In all patients, the CPH reconstruction resulted in nonunion or fracture. The patients then underwent a salvage procedure with delayed VFF reconstruction by a multidisciplinary surgical team consisting of a senior plastic surgeon and a senior orthopedic surgeon. All patients underwent preoperative angiogram assessment of the upper and lower extremities for artery and vein mapping.

The prior anterior incision of the upper extremity was used to access the humerus, and the dissection was carried down to the bone and the nonunion site. The broken fixation devices (plate and/or screws) were removed, and the nonunion site debrided down to bleeding bone. In preparation for the VFF, further exposure of the bone and vascular supply was performed.

The contralateral fibular length was measured and marked on the skin of the posterolateral leg. The fibula was isolated in the usual fashion. Harvested diaphysis fibular length averaged 12.4 cm (range = 10–16 cm) (Table 1). Osteotomies were performed superiorly and inferiorly at the measured length. The peroneal vessels were identified, dissected to provide a leash, and then carefully clipped and divided. Care was taken to preserve the tibial and peroneal nerves, as well as the anterior and posterior tibial arteries.

In the brachium, the fibular flap was placed in appropriate alignment alongside the clavicular graft and attached with bone screws to the proximal fragment of the clavicular graft and the distal fragment of the humerus. The appropriate alignment of the humerus was confirmed with fluoroscopy. The elbow was taken through a full range of motion to ensure the graft was not blocking elbow flexion or extension. In three patients (60%), allograft chips were mixed with demineralized bone matrix and placed at the nonunion site. Anastomosis was then performed between the peroneal artery of the flap and an artery of the recipient site (brachial artery, branch of the brachial artery, or circumflex humeral artery) in an end-to-end or end-to-side fashion. All venous anastomoses were performed in an end-to-end fashion with a venous coupler between the peroneal vein and a deep arm vein (Table 1). An implantable Doppler was typically placed around the vein and secured with a hemoclip to ensure a good venous signal.

At both surgical sites, adequate hemostasis was obtained, bulb suction drains placed, and incisions closed. A skin paddle was used if the overlying skin was heavily scarred or pulled excessively tight to provide more malleable soft tissue coverage. The arm was immobilized in a sling and swathe, and the ankle in a fiberglass splint.

## 3. Results

### 3.1. Demographics and Oncologic Characteristics

We identified five pediatric patients who underwent free VFF salvage reconstruction following failed CPH (Table 2). All of the flaps performed were osteocutaneous flaps. The study cohort consisted of two males (40%) and three females (60%) with a mean age of 8.4 years old (range = 5–10 years old) at the time of salvage surgery. The average time to salvage surgery from the CPH operation was 691.2 days (range = 287–1568 days). All patients presented with primary bone tumors and the proximal humerus was the involved site in all patients (*n* = 5). The pathologic diagnosis was osteosarcoma in four patients (80%), and Ewing sarcoma in one patient (20%). Two patients (40%) presented with metastatic disease. All patients received preoperative chemotherapy. One patient received additional postoperative radiation consisting of whole lung irradiation for 8 fractions. One patient had a patent foramen ovale, but otherwise, no patients had any significant past medical history. The mean follow-up time, defined as the time from the CPH procedure to the last documented clinic follow-up visit, was 3.7 years (range = 1.2–5.10 years) (Table 2). There was no local reoccurrence of the disease in any of the cases, and all patients were alive at the time of the study.

### 3.2. Complications

All patients experienced postoperative complications, representing a complication rate of 100% (Table 3). Of the five patients experiencing complications, three (60%) required immediate reoperation. One patient required revision of the venous anastomosis for thrombosis, one required partial skin paddle excision and advancement for partial necrosis, and one required surgical re-exploration for a lost signal from a dislodged Doppler and required only repositioning of the Doppler in the operating room. The two cases with postoperative complications which did not require reoperation (40%) included partial necrosis of the skin paddle treated conservatively (*n* = 1), and transient radial nerve palsy that resolved with conservative management (*n* = 1). There were no cases of total flap loss and no donor site complications. Subsequent postoperative radiographs revealed bilateral fibular bone integration at the recipient site. Since all flaps were osteocutaneous flaps, we were able to clinically assess the skin paddle viability. All the skin flaps remained viable, providing further reassurance of bone survival.

### 3.3. Bone Union and Functional Outcomes

**Figure 1:**
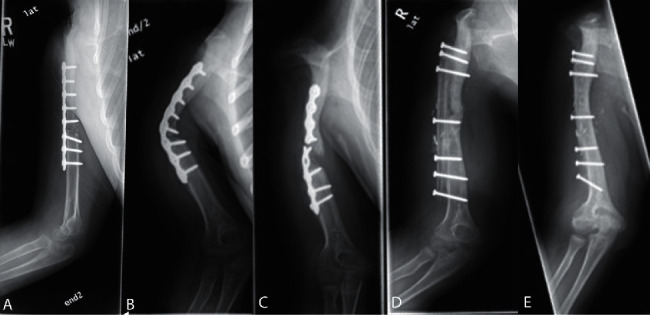
(a) 3 months after primary limb reconstruction with clavicula pro humero (CPH) procedure. (b) Nonunion at 9 months after original CPH procedure. (c) Subsequent fracture at 19 months after original CPH. (d) Secondary salvage procedure 24 months after CPH with onlay free vascularized fibular flap. (e) Union and remodeling of fibular flap 63 months after original CPH procedure.

Patients were followed up with radiographic imaging (Figure 1). Four patients (80%) achieved bony union at both ends of the fibula, with an average time to bony union of 3.7 months (range = 1.9–5.3 months) (Table 3). One patient (20%) experienced graft nonunion and fracture. This patient was scheduled for planned reoperation but was ultimately lost to follow-up prior to surgical intervention. Despite the occurrence of nonunion in one patient, all patients (*n* = 5) retained the salvaged limb. At the last clinic follow-up, all patients (100%) demonstrated intact sensation in the proximal upper extremity and well-healed surgical incisions. Additionally, all patients (100%) were able to perform regular activities of daily living with no pain complaints. Postoperative range of motion varied between patients depending on the muscular structures sacrificed during the initial tumor resection (Supplemental Table 2). Three patients (60%) were able to raise their hands to their forehead on physical exam. Active shoulder flexion for all patients ranged from 45 to 60°. Three patients (60%) had medical record documentation of full function and range of motion from the elbow distally with good hand strength; the distal range of motion of the other two patients was not specified.

## 4. Discussion

Primary bony tumors in the proximal humerus of pediatric patients are rare. The many reconstructive options compounded with challenging functional goals make the decision between the surgeon and parents for the best salvage method difficult. Regardless of the type of reconstruction, complications such as nonunion and fractures warranting further procedures can be common. In previously published systematic reviews, fractures were the most common complication of upper extremity reconstructions (35% and 11.7%) [21, 25]. The most common original limb salvage operations in the pediatric population include vascularized fibular flap (VFF) [26–29], endoprosthesis [30, 31], induced membrane techniques involving spacers [32, 33], and vascularized fibular epiphyseal transfers [29, 34]. Vascularized fibular epiphyseal transfers in particular have gained popularity as a first-line treatment option due to preserved longitudinal growth potential and adequate functional outcomes [29, 34]. Due to the expected risks of nonunion and fracture following limb salvage procedures regardless of the initial reconstructive method selected, it is essential to anticipate possible revision procedures and document successful salvage options for this population. One previously published manuscript on pediatric sarcoma reconstruction salvage was a case report (*n* = 1) documenting limb salvage with a combined clavicula pro humero (CPH) and VFF procedure following infection of the original megaprosthetic reconstruction [18]. The literature on pediatric proximal humerus reconstruction is limited, and elaboration on further procedures has rarely been described, aside from the previously mentioned case report. We aimed to fill this gap in the literature by reporting our experience with VFF salvage of failed CPH proximal humerus reconstruction.

All five (100%) of our patients experienced complications, with three (60%) needing further operations (patients 1, 3, and 4). The reoperations occurred in the immediate postoperative period. The high rate of complications may be attributed to the excessive local scar tissue and decreased blood flow from prior procedures as well as postradiation skin changes. Despite the high rate of postoperative complications, all patients retained the salvaged limb and were able to complete activities of daily living. Additionally, four patients (80%) achieved bony union by four months on average, which is consistent with the prior literature on VFF reconstruction of the proximal humerus [35–37].

### 4.1. Limitations

There are several limitations to this study, including the retrospective nature of the study which limits data collection. Another limitation is the small sample size. The rarity of bone sarcomas in any specific anatomic site in the pediatric population makes it difficult for institutions to gather significant case numbers. Our study further limits the number of cases as it focuses on our experiences with a secondary revisional procedure not well described in the literature. Another limitation is the average follow-up time of 3.7 years. Although several years of follow-up time is generally considered sufficient, in many pediatric reconstructive operations, the dissatisfaction with limb aesthetics or length discrepancies as patients grow can often drive further operations, which is not accounted for in our relatively short follow-up time. Additionally, our patients have not yet reached skeletal maturity, and the long-term durability of these reconstructions is yet uncertain.

## 5. Conclusion

Primary bone tumors in the pediatric population require wide surgical resection, and reconstruction often has high complication rates of nonunion or fractures that can warrant further procedures. A free VFF is a viable option for upper extremity salvage after previously failed reconstructions because it provides vascularized tissue to a scarred tissue bed and allows for the replacement or augmentation of large bony defects.

## Figures and Tables

**Table 1:** Free vascularized fibular flap (VFF) operative details.

Patient	Original defect size (cm)	Fibula length (cm)	Anastomosis	Artery anastomosis style	# of veins	Skin paddle size (cm)	Operative duration (min)	Estimated blood loss (mL)	Was allograft or DBM used at the nonunion site?	Mode of fixation
1^*∗*^	10.5	16	Peroneal to side branch of brachial	ETS	1	—	—	350	Yes	Screws

2^*∗∗*^	15	10	Peroneal to circumflex humeral	ETE	1	15 × 2.5	459	150	No	Screws

3	17	15	Peroneal to brachial	ETS	1	10 × 1.5	710	200	No	Plate (reused from original procedure), screws

4	15	11	Peroneal to side branch of brachial	ETS	1	15 × 2.5	497	50	Yes	Screws

5	8	10	Peroneal to circumflex humeral artery	ETE	1	15 × 3	462	375	Yes	Screws

^*∗*^Third revision surgery. ^*∗∗*^Fourth revision surgery. ETS: end-to-side; ETE: end-to-end; —: not available.

**Table 2:** Patient demographics and oncologic characteristics.

Patient	Sex	Age	Follow-up^*∗*^	Diagnosis	Location	Metastasis	Chemo	Radiation	Time to failure from CPH operation (days)^*∗∗*^
1	M	8 y 3 m	5 y 0 m	Osteosarcoma	Proximal humerus	No	Yes	No	611

2	F	9 y 1 m	3 y 4 m	Osteosarcoma	Proximal humerus	Yes	Yes	No	497

3	F	10 y 1 m	5 y 10 m	Ewing sarcoma	Proximal humerus	Yes	Yes	Yes (postoperative whole lung irradiation for 8 fractions)	493

4	M	10 y 2 m	3 y 2 m	Osteosarcoma	Proximal humerus	No	Yes	No	287

5	F	5 y 9 m	1 y 2 m	Osteosarcoma	Proximal humerus	No	Yes	No	1568

^*∗*^Follow-up defined as time from CPH procedure to last documented clinic follow-up visit. ^*∗∗*^Time to failure from CPH operation defined as days between CPH operation and secondary salvage procedure.

**Table 3:** Postoperative course and associated complications.

Patient	Postoperative complications	Treatment of complications	Reoperation details	Total # of associated reoperations	Time to osseous union (months)
1	Lost Doppler signal	Reoperation	Venous Doppler repositioning^a^	1	4.6
	Hypotension	Transfusion of 1 pRBC	—		—

2	Partial tissue necrosis	Conservative management	—	0	2.9

3	Skin paddle venous congestion	Reoperation	Skin paddle resection with advancement flap^b^		—
	Nonunion and fracture	Planned reoperation^*∗*^		2	—

4	Lost Doppler signal	Reoperation	Hematoma evacuation, flap vein thrombectomy and reanastomosis	1	5.3

5	Radial nerve palsy	Conservative management	—	0	1.9

^*∗*^Lost to follow-up before undergoing reoperation and achieving evidence of osseus union. ^a^Exploration of the right arm fibula flap revealed venous Doppler shifted in position but artery and vein were patent on exploration. ^b^Exploration revealed thrombosis of tiny perforator vessels with widely patent flap vessels. Skin paddle resected using Bovie, and an anterior skin subcutaneous tissue advancement flap 15 × 6 cm for right arm closure.

## Data Availability

The data are available from the authors upon reasonable request.

## Supplementary Materials

Supplementary Materials Supplemental Table 1: literature search of proximal humerus reconstruction with CPH procedure and associated complications. Supplemental Table 2: initial tumor resection and CPH reconstruction operative details.
