# Supplementary material for: Free Vascularized Fibula Salvage of Failed CPH in Pediatric Sarcoma Patients
Source: Sarcoma. 2022 May 9;2022:6240293. doi: 10.1155/2022/6240293 (PMC9110248; doi:10.1155/2022/6240293)
Supplement: Supplementary Materials — Supplemental Table 1: literature search of proximal humerus reconstruction with CPH procedure and associated complications. Supplemental Table 2: initial tumor resection and CPH reconstruction operative details. [file 6240293.f1.docx]

**Supplemental Table 1. Literature Search of Proximal Humerus Reconstruction with CPH Procedure and Associated Complications**

| Study | Year | Location | Cases | Age (mean) | Complication requiring surgical revision | Revision surgery |
| --- | --- | --- | --- | --- | --- | --- |
| Wozniak^12^ | 1996 | Poland | 3 | 13 | 1 (33%) | n/a |
| Ozaki^13^ | 2001 | Japan | 1 | 10 | 1 (100%) | Replacement with longer plates and VFF. |
| Rodl^14^ | 2002 | Germany | 15 | 18 | 10 (67%) | Complete clavicle removal (n=2), infection (n=4), replaced with cement spacer (n=1), prominent acromion with impending skin perforation (n=3) |
| Tsukushi^15^ | 2006 | Japan | 7 | 36 | 1 (14%) | Reconstruction plate pulled out of bone |
| Kitagawa^16^ | 2007 | Australia | 7 | 29 | 2 (29%) | Recurrence, DOD (n=2) |
| Nishida^17^ | 2008 | Japan | 2 | 16 | 1 (50%) | Prominence of acromion with skin perforation requiring flap coverage |
| Calvert^8^ | 2014 | USA | 4 | 6 | 2 (50%) | Onlay VFG |
| Clayer^18^* | 2015 | Australia | 1 | n/a | 0 |  |
| Barbier^19^ | 2016 | France | 8 | 13 | 4 (50%) | Resection, bone grafting, osteosynthesis (n=4) |
| Okimatsu^20^ | 2016 | Japan | 2 | 67 | 0 |  |

*Prior reconstruction with megaprosthesis.

**Supplemental Table 2: Initial Tumor Resection and CPH Reconstruction Operative Details**

| Patient | Tumor resection defect size (cm) | Sacrificed Structures | Mode of Fixation |
| --- | --- | --- | --- |
| 1 | 10.5 | Muscle: majority of deltoid  Nerve: axillary nerve | Plate |
| 2 | 15 | None | Plate |
| 3 | 17 | None | Plate |
| 4 | 15 | Muscles: pectoralis major insertion; anterior half of deltoid; long head of biceps  Nerve: none | Plate |
| 5 | 8 | Muscle: deltoid cuff  Nerve: None | Plate |
